# Stabilization and reversal of child obesity in Andalusia using objective anthropometric measures by socioeconomic status

**DOI:** 10.1186/s12887-018-1295-4

**Published:** 2018-10-11

**Authors:** José-Juan Sánchez-Cruz, I de Ruiter, J J Jiménez-Moleón, Ll García, Maria-Jose Sánchez

**Affiliations:** 1 Escuela Andaluza de Salud Pública, Cuesta del Observatorio 4, 18011 Granada, Spain; 2 Instituto de Investigación Biosanitaria ibs.GRANADA, Hospitales Universitarios de Granada/Universidad de Granada, Granada, Spain; 3 CIBER de Epidemiología y Salud Pública (CIBERESP), Madrid, Spain

**Keywords:** Child obesity, Trends, Epidemiology, Spain

## Abstract

**Background:**

Childhood obesity continues to be a significant public health issue worldwide. Recent national studies in Spain show a stable picture. However, prevalence and trends differ by socio-economic status, age, and region. We present the trend in childhood excess weight prevalence, aged 8–15 years, in Andalusia from 2011-2012 to 2015–2016 by socio-economic status.

**Methods:**

Using the cross-sectional Andalusian Health Surveys, objective anthropometric measures were taken for a representative sample of 8–15 year olds in Andalusia in 2011–2012 and 2015–2016. Prevalence and changes in prevalence of excess weight (overweight plus obesity) were calculated, using both the WHO and IOTF criteria, overall and for sex, age and three different indicators of SES.

**Results:**

Overall prevalence of excess weight decreased from 42.0% in 2011–2012 to 35.4% in 2015–2016. Overweight decreased from 28.2 to 24.2% and obesity from 13.8 to 11.2%. In 2011–2012 the prevalence of excess weight in boys was 46.0%and 37.9% in girls; in 2015–2016 the difference became significant with 41% of boys with excess weight compared with 30% in girls.

**Conclusions:**

Childhood excess weight prevalence in Andalusia has decreased slightly between 2011-2012 and 2015–2016. Notably, a decrease in obesity prevalence in girls aged 8–15 years was recorded. In 2011–2012 a social gradient for excess weight prevalence across three SES indicators was observed: in 2015–2016 this gradient disappeared. Nonetheless, prevalence remains too high.

## Background

Childhood obesity continues to be a significant and contemporary public health issue worldwide. In Spain, the prevalence of excess weight (overweight and obesity combined) has been reported as being well over 35% for children and adolescents, aged 8–17 [1]. Moreover, Spain ranks amongst the countries with the highest prevalence of overweight and obesity in the European Union [2, 3]. The high prevalence of excess weight being documented worldwide is concerning as childhood obesity results in both immediate and long term health consequences with risk profiles tracking into adulthood [4–6]. Child obesity has been shown to be a predictor of adult obesity and is also independently associated with adult cardiovascular outcomes and mortality [5, 7]: The first cause of mortality and one of the primary causes of hospitalisation in Spain [1, 4, 8]. During childhood, obesity affects health in all systems and in particular causes earlier onset and acceleration of cardiovascular disease and type-2 diabetes [6, 9, 10]. Moreover, child obesity has a permanent impact on adult health outcomes leading to large health and economic burdens for health systems [6]. Therefore, the need to intervene and implement primary prevention during childhood is imperative. The Spanish national administration - principally the Ministry of Health and Consumers Affairs and Ministry of Education - and the local and regional administrations have been trying to address and control the problem for a long time. The Strategy for Nutrition, Physical Activity and Prevention of Obesity (NAOS), which aims to improve diet and to encourage the regular practice of some physical activity by all citizens, paying special attention to prevention during childhood, is an example of these types of interventions [11]. The NAOS Strategy was drawn up by the Spanish Ministry of Health and Consumer Affairs in 2005. This Strategy also proposes carrying out epidemiological surveillance and monitoring of the obesity trend on a national scale, via the Autonomous Communities. Andalusia is a region of Spain with approximately 8.4 million inhabitants; similar in size to Switzerland or Austria.

Some countries, such as the United States or Australia, have reported a stabilisation or plateau in these trends at a national level [1, 4, 12–15]. Two recent national studies in Spain show an overall stable picture with some ups and downs; although one study did not use objective measures of height and weight or work, and neither study presented local data at a more provincial level [4, 16]. While this overall national stable trend is encouraging, studies have shown that excess weight prevalence and trends may differ by socio-economic status (SES) [17–20], age group [4, 13], and region [12, 15, 21].

Interventions aimed at combating childhood obesity may result in different outcomes in different demographic groups and therefore monitoring of the obesity epidemic by region, age and SES is imperative. In particular, lower socio-economic groups may not be benefiting from some interventions aimed at changing the obesogenic environment and may be causing increasing inequalities [15]. With the added impact of the recent socio-economic crisis, investigation of trends and prevalence by SES is even more important [18].

Given the need to carry out epidemiological surveillance of the obesity trend and the possible differences of tendency by social strata, we aim to present the change in overweight, obesity and excess weight prevalence in children and adolescents aged 8–15 years in Andalusia (South Spain) between 2011-2012 and 2015–2016 using objective anthropometric measures and to examine any changes in prevalence by age, sex and SES between these two periods.

## Methods

### Study design and population

The data analysed in this study stemmed from the Andalusian Health surveys carried out in 2011–2012 (from February 2011 to March 2012) and 2015–2016 (from March 2015 to February 2016). The Andalusian Health Surveys are designed to collect information related to the health and associated risk factors of the Andalusian population and the use of the health service system. The surveys have a non-experimental cross-sectional design. The random samples for the surveys were selected from the paediatric population resident in Andalusia.

The Andalusian Health Survey uses a probabilistic, stratified and multi-stage cluster sampling design with the following sampling stages: the municipalities, census sections, homes and individuals. Sample strata were determined based on the province and the urban/rural resident type. The sampling allocation for each stratum resulting from the province and habitat type was proportional to the existing population. A third stratification criterion was also used which was defined by the seasonal period of the interviews. The effective total sample size for the children in the interval 8–15 years was 796 in 2011–2012 and 688 in 2015–2016.

Data collection was carried out via a questionnaire completed during a personal interview with each individual selected for the study and the primary caregiver of the child (principally the mother in more than 75% of cases). The height and weight of children were measured at the time of interview. Height was measured in cm using a portable height rod: “Stature meter 2m. Model S-5002”. Weight was measured in kilos (100 g increments) using Tanita scales, Model UM076.

### Variables

To determine whether the children were obese or overweight, their body mass index and z scores corresponding to their age and gender (the child’s growth curve) were calculated. Children were subsequently classified as being normal weight, overweight or obese based on z scores as defined by the World Health Organisation (WHO) standards [22] and in addition using the IOTF (International Obesity Task Force) standard [23]; the two most widely used definitions. Children classified in the range for thinness or severe thinness were excluded from the analysis (a total of 19 cases were excluded from the analysis).

Data collected from the Andalusian Health surveys included different factors associated with SES, including parental educational level, employment status and social class. The variable “social class” was initially defined by the research group of the Spanish Epidemiology Society (SEE) [24]. However, due to the lack of data for some of the defined classes we regrouped “social class” into the following categories: I, II, III, IV, V and VI. These categories range from parents who have higher management roles with leading responsibilities (I), to parents with no paid employment (VI).

In this article we present the prevalences of the different levels of obesity according to two of the most widely accepted criteria: the WHO and the IOTF. Other studies have demonstrated differences in prevalences depending on the criteria used [25]; as such we present both to allow for better comparison with other studies and countries or regions. However, as the aim of this paper was not to compare in detail the two standards but rather to provide objective data on the prevalence of overweight and obesity as defined by the WHO standards, the main results will be presented using the WHO standards.

### Statistical analysis

Prevalence of excess weight (overweight and obesity), overweight and obesity were calculated for each survey year. For the categorical variables (age, sex, parental education, parental employment status, and social class), percentages of cases of overweight, obesity and excess of weight within each category are shown, as well as their 95% confidence intervals. As we used a complex sample design, with multi-stage probabilistic sampling, we applied the Bootstrap technique with 1000 Bootstrap samples to derive the variance of the estimators to calculate the confidence intervals and *p* values that are presented in the tables. Comparison of the proportions was done using the Pearson Chi-square test. The statistical package SPSS version 20 was used to perform the analysis.

## Results

Table 1 shows the measured prevalence overweight, obesity and excess of weight overall using the WHO definition, by age group, sex and SES variables in 2011–2012 and 2015–2016. Table 1 shows a statistically significant (*p* < 0.006) lower prevalence of excess weight (obesity and overweight combined) in the whole group, between 8 and 15 years inclusive, in 2015–2016 (35.4%) compared with 2011–2012 (42.0%) when we consider both sexes together. This significant difference, in the same sense, was also found in those with overweight (24.2% in 2015–2016 versus 28.2% in 2011–2012, *p* < 0.051). However, regarding obesity, although a lower prevalence is observed in 2015–2016 (11.2%) than in 2011–2012 (13.8%), this difference does not reach statistical significance (*p* < 0.094).

**Table 1:** Measured (objective) prevalence of overweight, obesity and excess weight overall using the WHO definition, by age group, sex and SES variables in 2011–2012 and 2015–2016

			2011-2012		2015-2016		*P value*
			% (95% CI)	N	% (95% CI)	n	
Total		Excess weight	42.0 (38.9–45.2)	398	35.4 (32.3–38.3)	344	0.006
		Overweight	28.2 (25.2–31.3)	267	24.2 (21.5–26.9)	235	0.051
		Obese	13.8 (11.6–16.0)	131	11.2 (9.2–13.1)	109	0.094
Age group (years)	8–10	Excess weight	51.5 (46.1–56.9)	172	43.8 (39.5–48.5)	172	0.024
		Overweight	28.4 (23.8–33.4)	95	26.2 (21.9–30.7)	103	0.484
		Obese	23.1 (18.7–27.4)	77	17.6 (13.6–21.5)	69	0.069
	11–13	Excess weight	43.5 (38.2–48.5)	148	35.8 (30.4–41.1)	113	0.043
		Overweight	30.9 (25.6–35.8)	105	26.9 (22.2–32.0)	85	0.259
		Obese	12.7 (9.0–16.1)	43	8.9 (5.9–12.4)	28	0.119
	14–15	Excess weight	28.5 (23.4–33.6)	78	22.5 (17.4–28.1)	59	0.12
		Overweight	24.5 (19.5–29.9)	67	17.9 (13.3–22.7)	47	0.068
		Obese	4.0 (1.8–6.4)	11	4.6 (2.2–7.5)	12	0.756
Sex	Boys	Excess weight	46.0 (41.6–50.6)	222	41 (36.5–45.4)	196	0.121
		Overweight	31.3 (27.3–35.2)	151	25.5 (21.6–29.8)	122	0.04
		Obese	14.7 (11.5–18.0)	71	15.5 (12.3–18.7)	74	0.735
	Girls	Excess weight	37.9 (33.4–42.2)	176	30.0 (26.1–34.1)	148	0.011
		Overweight	25.0 (21.0–29.2)	116	22.9 (19.1–26.6)	113	0.466
		Obese	12.9 (9.9–16.2)	60	7.1 (4.9–9.3)	35	0.005
Highest Education Level	Primary and compulsory secondary education	Excess weight	43.2 (39.4–47.1)	278	37.1 (33.2–41.1)	221	0.03
		Overweight	27.2 (23.6–30.8)	175	24.2 (20.9–27.9)	144	0.238
		Obese	16.0 (13.0–18.9)	103	12.9 (10.3–15.6)	77	0.134
	Completed Secondary education	Excess weight	42.5 (35.0–50.5)	74	30.6 (24.9–36.5)	68	0.026
		Overweight	32.8 (25.5–40.1)	57	22.1 (17.0–27.7)	49	0.024
		Obese	9.8 (5.5–14.5)	17	8.6 (5.0–12.3)	19	0.68
	Tertiary level	Excess weight	35.4 (27.1–43.4)	46	35.1 (27.1–43.1)	53	0.961
		Overweight	26.9 (19.7–34.2)	35	26.5 (20.1–33.8)	40	0.928
		Obese	8.5 (3.9–13.6)	11	8.6 (4.5–13.4)	13	0.973
Employment status	Working	Excess weight	40.9 (36.1–45.7)	179	35.9 (32.0–40.2)	167	0.116
		Overweight	26.9 (22.5–31.1)	118	24.5 (20.7–28.4)	114	0.401
		Obese	13.9 (10.8–17.2)	61	11.4 (8.7–14.4)	53	0.234
	Unemployed	Excess weight	44.0 (37.7–50.7)	107	36.4 (30.5–42.0)	103	0.076
		Overweight	29.2 (23.6–35.4)	71	23.7 (19.1–29.3)	67	0.16
		Obese	14.8 (10.5–19.9)	36	12.7 (9.0–16.2)	36	0.489
	Household activities	Excess weight	42.1 (35.9–48.5)	104	33.0 (25.7–40.6)	59	0.061
		Overweight	29.6 (24.1–36.0)	73	24.6 (18.7–31.5)	44	0.244
		Obese	12.6 (8.4–16.7)	31	8.4 (4.8–12.9)	15	0.158
Social class based on the occupation the head of the household	I y II (Directors and Managers)	Excess weight	33.7 (24.7–43.8)	31	38.5 (28.8–48.3)	37	0.492
		Overweight	25.0 (15.9–34.1)	23	28.1 (18.8–37.1)	27	0.63
		Obese	8.7 (3.1–14.8)	8	10.4 (4.8–17.0)	10	0.69
	IIIa, IIIb, IIIc (Administration, supervisors, self-employed)	Excess weight	39.9 (32.7–48.2)	65	26.8 (20.4–33.5)	45	0.012
		Overweight	28.2 (21.1–34.9)	46	19.1 (13.4–25.3)	32	0.042
		Obese	11.7 (6.9–17.0)	19	7.7 (4.1–12.2)	13	0.23
	IVa, IVb (Skilled technical Workers)	Excess weight	43.8 (38.3–49.7)	144	37.2 (31.8–42.0)	133	0.078
		Overweight	28.3 (23.7–33.0)	93	24.0 (19.6–28.3)	86	0.207
		Obese	15.5 (11.5–19.5)	51	13.1 (9.6–16.9)	47	0.376
	V y VI (Skilled manual workers + non-skilled workers)	Excess weight	44.6 (38.1–51.5)	95	37.2 (30.6–43.8)	79	0.125
		Overweight	28.6 (22.7–34.6)	61	24.5 (18.9–30.5)	52	0.339
		Obese	16.0 (11.3–20.7)	34	12.7 (8.0–17.5)	27	0.344

In the 2011–2012 survey, prevalence of excess weight in 8–10 year olds was 51.5%, 43.5% in the 11–13 year olds group, and 28.5% in the 14–15 year olds group. A similar significant downward change was seen in 2015–2016 (43.8%, 35.8% and 22.5%, respectively). Between 2011 and 2012 and 2015–2016, in the three age groups a general downward change can be seen (with values that approximately range between 3 and 7 absolute percentage points) for excess weight as well as for the overweight and obese categories (except when we compare both time periods for the 14–15 year old age group where the prevalence is observed to remain almost the same, but a too small sample size is noted).

With regards to the sex variable, in 2011–2012 the prevalence of excess weight in boys was 45.96, higher than the 37.9% in girls. In 2015–2016 the difference became significant (41% of boys classified as having excess weight compared with 30.0% in girls). Comparing the time periods studied in the two health surveys, we observe that in 2015–2016 the prevalence of excess weight, obesity and overweight was lower for both girls and boys compared to 2011–2012; with the exception of obesity in boys that barely reaches a non-significant percentage point increase.

Table 2 shows the above prevalences using the IOTF definition for comparison. Overall the measured prevalence was lower using the IOTF cut-offs compared with those using the WHO definition. Nonetheless, the same general pattern was seen, with a significant downward change; for example, in excess weight from 33.4% in 2011–2012 to 27.6% in 2015–2016. Overweight prevalence decreased from 24.6 to 21.3%. Obesity decreased from 8.9% in 2011–2012 to 6.2% in 2015–2016. On stratifying for the variables age and sex the results show a similar pattern to those seen when using the WHO criteria.

**Table 2:** Measured (objective) prevalence of overweight, obesity and excess weight overall using the IOTF definition, by age group, sex and SES variables in 2011–2012 and 2015–2016

			2011		2015		*P* value
			% (95% CI)	n	% (95% CI)	n	
Total		Excess weight	33.4 (30.3–36.7)	317	27.5 (24.7–30.4)	267	0.006
		Overweight	24.6 (21.9–27.6)	233	21.3 (18.7–24.0)	207	0.078
		Obese	8.9 (7.0–10.5)	84	6.2 (4.7–7.7)	60	0.022
Age group (years)	8–10	Excess weight	42.2 (36.6–47.2)	141	32.3 (27.7–37.0)	127	0.01
		Overweight	27.0 (22.4–32.1)	90	22.9 (19.0–27.2)	90	0.22
		Obese	15.3 (11.3–19.4)	51	9.4 (6.6–12.3)	37	0.016
	11–13	Excess weight	33.2 (28.3–38.4)	113	27.5 (22.8–32.4)	87	0.102
		Overweight	25.6 (20.9–30.8)	87	22.8 (18.3–27.4)	72	0.396
		Obese	7.7 (5.0–10.4)	26	4.8 (2.5–7.4)	15	0.099
	14–15	Excess weight	23.0 (17.9–27.7)	63	20.2 (15.5–25.4)	53	0.438
		Overweight	20.4 (16.0–25.2)	56	17.2 (12.5–21.6)	45	0.335
		Obese	2.6 (0.8–4.7)	7	3.1 (1.2–5.5)	8	0.728
Sex	Boys	Excess weight	34.4 (30.4–38.7)	166	30.3 (26.2–34.3)	145	0.182
		Overweight	26.1 (22.2–30.2)	126	23.0 (19.4–26.8)	110	0.269
		Obese	8.3 (5.9–10.9)	40	7.3 (5.0–9.8)	35	0.58
	Girls	Excess weight	32.5 (28.1–36.7)	151	24.8 (20.7–28.4)	122	0.009
		Overweight	23.0 (18.9–26.8)	107	19.7 (16.2–23.2)	97	0.209
		Obese	9.5 (6.9–12.5)	44	5.1 (3.2–6.9)	25	0.012
Highest Education Level	Primary and compulsory secondary education	Excess weight	34.9 (31.2–38.6)	225	29.6 (26.1–33.4)	176	0.04
		Overweight	24.8 (21.7–27.9)	160	21.7 (18.4–25.0)	129	0.158
		Obese	10.1 (7.9–12.6)	65	7.9 (5.8–10.1)	47	0.176
	Completed Secondary education	Excess weight	33.9 (27.4–40.5)	59	22.5 (17.3–27.8)	50	0.012
		Overweight	25.9 (19.3–32.3)	45	19.4 (14.2–24.7)	43	0.134
		Obese	8.1 (4.1–13.1)	14	3.2 (0.9–5.7)	7	0.053
	Tertiary level	Excess weight	25.4 (17.9–32.8)	33	26.5 (19.7–34.4)	40	0.834
		Overweight	21.5 (14.1–28.7)	28	22.5 (16.0–29.3)	34	0.844
		Obese	3.9 (0.8–7.6)	5	4.0 (1.3–7.6)	6	0.956
Employment status	Working	Excess weight	32.2 (27.9–36.6)	141	29.3 (24.8–33.2)	136	0.355
		Overweight	22.2 (18.3–26.3)	97	22.6 (19.0–26.2)	105	0.869
		Obese	10.1 (7.2–12.9)	44	6.7 (4.5–8.9)	31	0.064
	Unemployed	Excess weight	35.0 (29.0–40.9)	85	27.2 (21.6–32.9)	77	0.076
		Overweight	25.1 (20–30.8)	61	20.1 (15.4–24.8)	57	0.172
		Obese	9.9 (6.1–13.9)	24	7.1 (4.2–10.2)	20	0.254
	Household activities	Excess weight	42.1 (35.6–48.1)	85	33.0 (26.3–39.8)	43	0.046
		Overweight	28.3 (22.9–34.0)	70	20.7 (15.2–26.8)	37	0.072
		Obese	6.1 (3.1–9.1)	15	3.4 (1.1–6.2)	6	0.195
Social class based on the occupation the head of the household	I y II (Directors and Managers)	Excess weight	29.4 (20.3–39.3)	27	27.1 (18.6–36.1)	26	0.732
		Overweight	23.9 (16.1–33.8)	22	21.9 (14.0–31.2)	21	0.74
		Obese	5.4 (1.1–11.0)	5	5.2 (1.1–10.2)	5	0.945
	IIIa, IIIb, IIIc (Administration, supervisors, self-employed)	Excess weight	31.3 (25.0–38.7)	51	19.1 (13.0–25.7)	32	0.008
		Overweight	23.3 (16.4–30.0)	38	16.7 (10.9–22.3)	28	0.153
		Obese	8.0 (4.2–12.6)	13	2.4 (0–5.1)	4	0.022
	IVa, IVb (Skilled technical Workers)	Excess weight	34.4 (29.2–39.5)	113	30.7 (26.3–35.7)	110	0.32
		Overweight	23.4 (18.5–28.4)	77	22.1 (17.9–26.1)	79	0.655
		Obese	10.9 (7.7–14.6)	36	8.7 (5.8–11.7)	31	0.311
	V y VI (Skilled manual workers + non-skilled workers)	Excess weight	36.2 (29.6–42.7)	77	30.2 (23.8–36.7)	64	0.195
		Overweight	25.8 (20.1–32.2)	55	23.1 (17.0–28.8)	49	0.517
		Obese	10.3 (6.5–14.5)	22	7.1 (3.8–10.7)	15	0.235

Table 1 shows the prevalence of overweight, obesity and excess weight by parental education level, parental employment status and parental SES using the WHO criteria. Overall lower prevalences were observed in 2015–2016. In both the education level variable and the employment status variable a downward change in prevalence was observed for excess weight, overweight and obesity. This pattern was also seen for the social class variable, with the exception of the “Social Class I” category which experienced stabilization or a slightly higher - but not statistically significant - prevalence of overweight, obesity and excess weight in 2015–2016.

In the highest education level variable, the lowest prevalence of excess weight in 2011–2012 was observed in children whose parents had tertiary level education. In contrast, in 2015–2016 the lowest prevalence of excess weight is seen in those whose parents completed secondary education. For this variable, between the time periods 2011–2012 and 2015–2016 there was downward change for all three weight groups (overweight, obesity and excess weight) in all categories, with the exception of the “tertiary level” category, in which the prevalence remains level.

In both the period 2011–2012 and 2015–2016 the three categories of the “Employment status” variable (working, unemployed, household activities) show similar levels of excess weight, obesity and overweight. Nevertheless, in 2015–2016 compared to 2011–2012, in all three employment status categories a lower prevalence is seen for each weight status group, although no statistical significance is reached.

With regards to the socio-economic variable based on the occupation of the head of the household, in the period 2011–2012 an upward gradient for obesity, overweight and excess weight is seen corresponding with increasing social class (from class I to VI). In contrast, in 2015–2016, the prevalences are very similar in all categories of social class, except in social class III which showed lower prevalences.

## Discussion

This study presents comparison of overweight and obesity in children and adolescents in Andalusia (South Spain) between 2011-2012 and 2015–2016 using objective anthropometric measures. An overall statistically significant decrease in excess weight was observed, decreasing from 42.0% in 2011–2012 to 35.4% in 2015–2016. This decrease is independent of the criteria used to define overweight and obesity, WHO or IOTF criteria, and is based on a representative sample and objective, physical, measures. When analyzing by weight status, age, sex and SES, a decrease in the prevalence of excess of weight can be observed for most groups, although the magnitude of this decrease is different for each.

Older adolescents had a lower prevalence of excess weight compared with younger children in both survey years; and a widening gap between boys and girls suffering from obesity was found. For SES and education level of the parents, the prevalence of excess weight surprisingly becomes equal between levels. While the prevalence of excess the weight decreased in lower and middle classes, this decrease was not observed in higher classes.

The finding of a plateau or slight downward change is consistent with national and other regional studies in Spain, as well as findings in other developed countries [4, 13, 16, 26–29]. Internationally, countries reporting a stable child obesity prevalence include Australia, England, Denmark, Sweden, Greece and the USA [14, 28, 30]; with Switzerland recording a decrease in child obesity prevalence [30]. Other studies in Spain have shown a recent decrease in excess weight [1, 16, 18]. However, the data used in these studies undertaken in Spain, are prior to 2015, they usually work with samples of younger children [1, 16, 18], they do not usually use objectives measures of weight and height [18], or they work with local samples used for other purposes such as clinical trials [18]. The ALADINO study is one example which has shown a recent decrease in excess weight prevalence in 6–9 year olds in Spain using objective measures [16]. Another study from central Spain similarly showed the development of a plateau in obesity prevalence rates, although this study is based on data from two cluster randomized trials aimed to evaluate the effectiveness of leisure-time physical activity on the prevention of childhood obesity and included population-based samples of children attending public schools in the Castilla-La Mancha region, Spain [18].

While these downward or stable tendencies in the prevalence rate of child obesity in Andalusia are a positive finding, they remain too high and require further intervention. Many theories have been postulated to explain the stabilisation of child excess weight in different countries and sub-populations worldwide. Obesogenic factors entail a complex interaction between biology, environment and behaviour [15]: potentially we have reached an equilibrium or saturation level in obesogenic factors [14, 18]. Alternatively, population level intervention programmes may be showing a positive effect. In Spain, the national level programme NAOS was introduced in 2005 [11], more recently the introduction of the Food Safety and Nutrition Act [31], a school based program PERSEO, and in Andalusia specifically a Child Obesity Plan was designed to promote healthy lifestyle behaviours. The Andalusian Child Obesity plan included action to promote the prevention and early detection and treatment of child obesity in Andalusia via health professionals in public medical centres [32]. These programs seem to have had an effect, although the benefits have not been similar in the different population groups and the figures reached are still inadmissible.

The decrease in prevalence with increasing age stayed the same between the two survey years. This lower prevalence amongst adolescents is a pattern consistent with other studies in Spain and also international studies [1, 4, 33–35]. The lower prevalence in older adolescents may be due to different effects: 1) cohort effect, 2) incidence vs. persistence, and 3) age effect. The stable prevalence of excess weight in the older age group between the two surveys makes a cohort effect less likely as those that were in the younger group in 2011–2012 are categorised in the older adolescents in 2015–2016. Most likely we are seeing a combination of incidence vs. persistence and an age effect with changing physiological, environmental, social and behavioural aspects. In Spain, as in this study, few young children who suffer from excess weight continue to do so into the teenage years [4]. With increasing age and growing independence, social and culture pressure and norms may have increasing influence on body weight and appearance. Studies have shown differences in diet and activity-related behaviours between young children and teenagers [36].

In 2011–2012 no difference in excess weight prevalence was seen between boys and girls. However obesity prevalence was lower in girls in 2015–2016 compared with 2011–2012, resulting in an increased difference in prevalence with boys in 2015–2016. This difference was not seen for overweight prevalence. The sex differences in trends observed are consistent with other Spanish studies [2, 4, 12]. Differing trends between sexes have also been observed in European countries including England, Germany and Sweden [26, 29, 37]. In comparison, a recent study in the USA did not find any differences between boys and girls [13]. In older children and adolescents these differences may potentially be contributed to by physiological differences such muscle mass. Additionally, studies have shown sex differences in weight-related behaviours [36, 38]. Weight perception should also be taken into account and potentially female adolescents experience more social pressure on appearance and beauty norms [34, 39].

Lower socio-economic classes have been consistently associated with higher prevalence of excess weight [19, 40]; this is generally thought to be partially due to by differing weight-related behaviours by SES [41]. However the relationship between SES and obesity is not consistent between countries or regions, is complex and influenced by the proxy or indicator used [19]: Social class or income may influence behaviour due to purchasing power or available opportunities and parental education level tends to correspond with health literacy [24]. In our study we presented estimates for parental education, employment status and social class. Although these factors correlate well with each other, they are not completely interchangeable [24]. In light of the recent financial crisis it is possible that their associations with obesity have been affected; for example, parental education level may no longer be as closely associated with income or employment status. Furthermore, international studies showing stabilisation trends in childhood obesity have shown that not all social classes have been affected equally increasing social inequalities in excess weight [15, 20, 37, 40]. In comparison, our results show no significant social gradient for excess weight prevalence, for none of the three SES indicators used; although a tendency towards a social gradient was seen in 2011–2012 which reduced in 2015–2016. This may be due to Andalusian social policies that provide a high level of protection to those in lower social classes and may reduce any associations or gradient [1]. Along these lines, a study in central Spain did find a social gradient which reversed over time [18], suggesting regional differences even within Spain. The reason for this regional discrepancy is beyond the scope of this study, but may be due to differing regional child obesity or social protection policies. Regardless of the results observed, the frequency of excess weight continues to be very high in Andalusia and new attempts at improvement are necessary. It is possible that a saturation effect may be observed in higher social classes and so it is difficult to further lower the prevalence of overweight in these groups. However, this is not an excuse to not intervene.

Finally, this study has a number of weaknesses which should be mentioned: 1) The results are cross-sectional in nature and therefore we cannot infer any causal relationships and any associations observed may go in either direction or be bi-directional in nature; 2) The sample population is also a region of Spain and results may not be able to be applied to other regions or populations. However, this study also has numerous strengths: 1) In particular the large sample size that was obtained, which was representative of the target population; 2) Anthropometric measures were also collected objectively avoiding the possibility of over- or under-estimating of obesity as occurs in studies using parental-reported height and weight.

## Conclusions

Prevalence of excess weight amongst children and adolescents aged 8–15 years in Andalusia has shown an overall slight downwards tendency between 2011-2012 and 2015–2016. A significant decrease in obesity prevalence in girls was recorded with an increased gender gap seen in obesity prevalence in 2015–2016 with respect to 2011–2012. In 2011–2012 a social gradient for excess weight prevalence was observed (obesity prevalence higher with a lower social class). However, in 2015–2016 this gradient disappeared, decreasing and equalising the prevalence in all social class. Using both the WHO and the IOTF cut-offs resulted in the same conclusions, although the prevalences in the different excess weight categories are higher when using the WHO criteria. Nonetheless, excess weight prevalence remains too high and need further intervention.

## Abbreviations

IOTF: International Obesity Task Force
SES: Socio-economic status
WHO: World Health Organisation
